# The Influence of Climate, Soil and Pasture Type on Productivity and Greenhouse Gas Emissions Intensity of Modeled Beef Cow-Calf Grazing Systems in Southern Australia

**DOI:** 10.3390/ani2040540

**Published:** 2012-10-01

**Authors:** Matthew J. Bell, Brendan R. Cullen, Richard J. Eckard

**Affiliations:** Melbourne School of Land and Environment, University of Melbourne, VIC 3010, Australia; E-Mails: bcullen@unimelb.edu.au (B.R.C.); rjeckard@unimelb.edu.au (R.J.E.)

**Keywords:** beef cow-calf, modeling, location, greenhouse gas emissions, grazing system

## Abstract

**Simple Summary:**

Livestock production systems and the agricultural industries in general face challenges to meet the global demand for food, whilst also minimizing their environmental impact through the production of greenhouse gas (GHG) emissions. Livestock grazing systems in southern Australia are low input and reliant on pasture as a low-cost source of feed. The balance between productivity and GHG emission intensity of beef cow-calf grazing systems was studied at sites chosen to represent a range of climatic zones, soil and pasture types. While the climatic and edaphic characteristics of a location may impact on the emissions from a grazing system, management to efficiently use pasture can reduce emissions per unit product.

**Abstract:**

A biophysical whole farm system model was used to simulate the interaction between the historical climate, soil and pasture type at sites in southern Australia and assess the balance between productivity and greenhouse gas emissions (expressed in carbon dioxide equivalents, CO_2_-eq.) intensity of beef cow-calf grazing systems. Four sites were chosen to represent a range of climatic zones, soil and pasture types. Poorer feed quality and supply limited the annual carrying capacity of the kikuyu pasture compared to phalaris pastures, with an average long-term carrying capacity across sites estimated to be 0.6 to 0.9 cows/ha. A relative reduction in level of feed intake to productivity of calf live weight/ha at weaning by feeding supplementary feed reduced the average CO_2_-eq. emissions/kg calf live weight at weaning of cows on the kikuyu pasture (18.4 and 18.9 kg/kg with and without supplementation, respectively), whereas at the other sites studied an increase in intake level to productivity and emission intensity was seen (between 10.4 to 12.5 kg/kg without and with supplementary feed, respectively). Enteric fermentationand nitrous oxide emissions from denitrification were the main sources of annual variability in emissions intensity, particularly at the lower rainfall sites. Emissions per unit product of low input systems can be minimized by efficient utilization of pasture to maximize the annual turnoff of weaned calves and diluting resource input per unit product.

## 1. Introduction

Livestock production systems and the agricultural industries in general face challenges to meet the global demand for food, whilst also abating greenhouse gas (GHG) emissions. Livestock production is one of the major contributors to GHG production globally [1,2]. In Australia, agriculture is a major source of methane (CH_4_) and nitrous oxide (N_2_O) emissions, which account for 58.9% and 85.9%, respectively, of the net national emissions of these GHGs [3]. 

Livestock grazing systems in southern Australia are reliant on pasture as a low-cost source of energy and nutrients. Typically, beef cattle grazing systems have low resource input; whereby nitrogen (N) inputs largely come from legume fixation and supplementary feed (in the form of grass silage, hay or grain) if used to fill gaps in pasture supply. Southern Australia has variable climate and pasture systems that need to be managed to cope with inter-annual variability [4]. Grazing systems in this region are largely dependent on the productivity of perennial grass and clover pastures. Climate, soil type and pasture species influence the quality, quantity and reliability of the pasture produced [5]. The productivity of grazing systems, such as live weight gain in cattle, is strongly related to the availability of newly-grown and digestible plant material [6], which can be influenced by pasture management. A winter-active and drought tolerant C3 pasture species such as phalaris (*Phalaris aquatica*) is common in Mediterranean climatic zones in Australia. In comparison, less digestible C4 subtropical grasses like kikuyu (*Pennisetum clandestinum*) are also commonly found in coastal areas of Australia. 

Biophysical models can simulate the interactions between the climate, soil properties and grazing animals to predict the pasture produced at a given location. Process-orientated models have been shown [7,8] to be important when comparing regional differences in GHG emissions, particularly when predicting N_2_O emissions from soils under different land use. The authors of both studies suggested that for national inventory calculations, more accurate emission factors for soil processes could be obtained based on models that relate soil processes dynamically. One such model is the Sustainable Grazing Systems (SGS) Pasture Model used in this study, which can simulate carbon (C) and N fluxes within the soil. There are few published studies [9,10] that have investigated the influence of geographic location on the relationship between animal productivity, carrying capacity of the system and associated greenhouse gas emission intensity. 

This study tests the hypothesis that increased productivity can minimize the GHG emissions intensity of beef cow-calf grazing systems and explores the influence of different climate, soil and pasture types in southern Australia on predictions of (1) the productivity, represented by carrying capacity and the amount of calf live weight at weaning per hectare and (2) the greenhouse gas emissions intensity per hectare and per kg calf live weight at weaning from beef cow-calf grazing systems with and without supplementary feed given. The SGS Model was used to simulate these interactions between pasture species, climate, soil properties and the grazing animal.

## 2. Experimental Section

### 2.1. Sites Simulated

The SGS Program, which investigated various aspects of grassland productivity and sustainability in southern Australia between the years 1996 to 2001 [11], provided data for the development and testing of the model [12,13]. Four sites were chosen (Table 1) to represent grazing systems with different climatic zones and soil types, which were: Albany [14], Dookie (for this location the SGS site at Ruffy was used for comparison [15]), Vasey [16] and Wagga Wagga [4,13]. Regionally specific dryland (*i.e.*, rainfed) perennial pasture systems were modelled at each site simulated. 

**Table 1:** Sites studied, their location, climate, pasture species and soil type [17] and observed average daily minimum and maximum temperatures and annual rainfall (from the years 1971 to 2000).

Site	Location	Lat., Long.	Climate	Pasture species	Soil type	Temp Min (°C)	Temp Max (°C)	Rainfall (mm)
Albany	SW Western Australia	−34.90, 117.80	Temperate	Kikuyu (*Pennisetum clandestinum*), subterranean clover (*Trifolium subterranean*)	Petroferric brown sodosol	10.7	20.5	780
Dookie	N Victoria	−36.37, 145.70	Mediterranean	Phalaris (*Phalaris aquatic*), subterranean clover, annual ryegrass (*Lolium rigidum*)	Vertic calic red chromosol	8.1	20.3	576
Vasey	SW Victoria	−38.25, 145.93	Mediterranean	Phalaris, subterranean clover	Yellow sodosol	7.4	19.1	624
Wagga Wagga	S New South Wales	−41.08, 145.77	Mediterranean	Phalaris, subterranean clover, annual ryegrass	Red chromosol/leptic tenosol	9.6	22.2	549

### 2.2. Whole System Model

A dynamic model incorporating climate data, soil properties, pasture species, livestock and management was used to describe the whole farm system on a daily time step. The SGS Model (documentation and model available at http://www.imj.com.au) has been shown to adequately simulate pasture-based systems with different climates, soil types and pasture species [13,18,19,20]. These studies have tested the predictive ability of the pasture module, which is sensitive to changes in the environment. The productivity of the grazing animal is dependent on the user’s definition of the energy requirement of a single animal, as described below, and the energy supplied by feed. 

The climate data inputs include minimum and maximum temperature (°C), rainfall (mm), solar radiation (MJ/m^2^) and vapor pressure (kPa). Simulations used historical climate data from the SILO database [21] from the years 1961 to 2000 (Table 1). 

### 2.3. Cow and Calf Grazing Systems at Each Site

**Figure 1:**
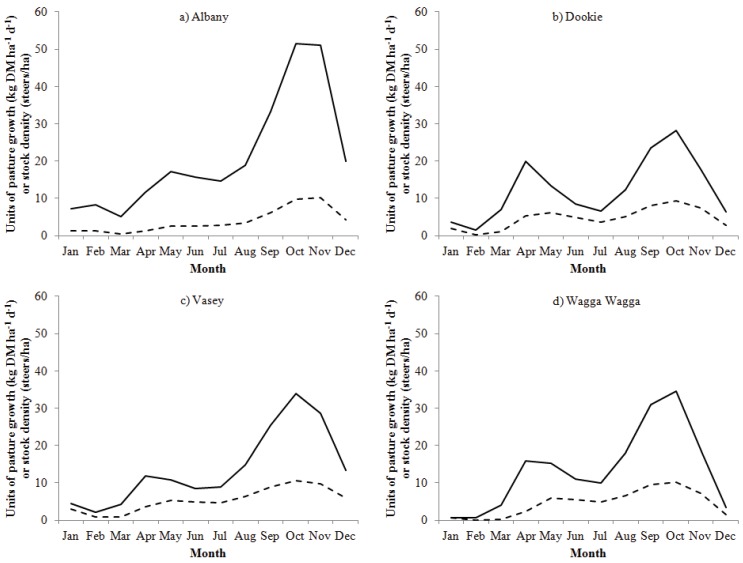
Predicted average daily pasture growth (─; kg DM ha^−1^ d^−1^) and stock density (- -; steers/ha) from January to December for the years of 1971 to 2000 at (**a**) Albany, (**b**) Dookie, (**c**) Vasey and (**d**) Wagga Wagga.

The SGS Model was used to predict the long-term carrying capacity of the pasture base, as these data were not available from the sites simulated. The model does not allow adjustment of cows with offspring on a daily time-step, therefore the carrying capacity was estimated by adjusting the number of steers grazing a one hectare paddock on a daily time-step, which in this case the pasture biomass was grazed to 0.5 t DM ha^−1^ for the phalaris pasture and 1 t DM ha^−1^ for the kikuyu pasture when pasture was available and then animals were removed. The kikuyu pasture was grazed less intensively due to its rhizomatous and stoloniferous growth [22]. Steers were assumed to maintain a 500 kg live weight and have a maintenance energy requirement of 67.5 MJ/d (0.58 × 500^0.75^ MJ d^−1^ with an additional 10% added for energy expended by activity). The adjusted stocking rate depended on pasture production and its quality as shown in Figure 1. The average number of steers/ha was converted to cows/ha for 30 year simulation runs by assuming a cow with a single calf (referred to as cow + calf from hereon) has a metabolizable energy (ME) requirement of 1.5 times that of a steer with a 500 kg live weight [23]. 

Throughout the year cows grazed four paddocks in a grazing system that optimized pasture intake/ha at each site. Relative comparisons could therefore be made between the sites studied, as the management of the grazing systems simulated at each site was the same. The timing of calving (the first day of the month) was set to coincide with when the cows annual pasture intake/ha was greatest, whilst also minimizing the need for supplementary feed. Cows calved at the start of July at each site (Figure 1), with an assumed 100% calving percentage and no calf mortality. The maximum growth rate of the calf was set at 0.6 kg d^−1^ from birth until weaning at 120 days of age, the point at which the calf was removed from the system. The target live weight of a calf at weaning was 100 kg. Cows and their calves were allowed to move between the four paddocks so that the available pasture biomass was maintained between 0.5 to 2.5 t DM ha^−1^ for a phalaris pasture and 1 to 2.5 t DM ha^−1^ for the kikuyu pasture. Any surplus pasture was cut to maintain a vegetative and palatable pasture for the grazing animal, at a time when pasture growth can be high, particularly in September to November (Figure 1). When the pasture biomass reached 2.5 t DM ha^−1^ it was assigned for cutting, which occurred at 3.5 t DM ha^−1^. 

The animal module describes the animal’s energy requirements for maintenance (defined as 0.58 × live weight^0.75^ MJ d^−1^), feeding activity, pregnancy, lactation and live weight change (see documentation at http://www.imj.com.au for a full description). The model assumes that the animal will try to eat enough feed to meet its ME requirement, with ME intake constrained by the ME content of available feed and a constraint on the animal’s maximum DM intake of 16.9 kg [24]. The ME in this study was for the cow + calf, with any limitation on calf growth being due to a restriction on ME consumption. 

Simulations were run with and without supplementary feed being given to the cow + calf. Therefore the diet consisted of either solely pasture or pasture and supplement. When there was no feed available, the cow and its calf would be removed from the system. When the diet was supplemented, grass silage and/or bought-in low quality grain were assumed to be fed to meet the animal’s ME requirement and maintain the live weight of the cow above 450 kg and lactation requirement. The model does not predict the quality of grass silage produced on the farm, which is being fed back to the animal. Therefore an assumption had to be made about the quality of the supplementary feed, which was fixed at 10.5 MJ kg DM^−1^ for ME content and 2.5% nitrogen for sites with a phalaris pasture system and an ME content of 10 MJ kg DM^−1^ and 2.0% nitrogen for the site with a kikuyu pasture system. Any surplus grass was cut and removed or made available for feed in the form of grass silage; with 10% wastage assumed during the cutting process. Due to the modeling approach used, the grass silage was only available as feed during the twelve months after being harvested (after which time it was removed from the system), which is a limitation of the model. 

Nitrogen inputs within the grazing system came from plant residues, dung, urine and nitrogen fixation by legumes, with no synthetic fertilizer used. The model is based on the assumption that nitrogen, water and temperature can all limit pasture growth. The simulations assumed sufficiency of nutrients other than nitrogen and water. 

### 2.4. Grazing System Greenhouse Gas Emissions

The CO_2_-eq. emissions associated with each site for the cow + calf over a year were predicted. Total system CO_2_-eq. emissions were calculated up to the farm-gate using the same approach to account for total production system emissions as that used by [25] when assessing dairy systems, except the present study did not include the GHG emissions produced by replacement animals. Emissions from the grazing system were assessed by CO_2_-eq. emissions per hectare and per kg of total calf live weight at weaning (the functional units as defined by [26]). The CO_2_-eq. emissions were calculated using conversion factors from CH_4_ and N_2_O to CO_2_-eq. emissions of 21 and 310 (for a 100 year time horizon) respectively [3]. The sources of CO_2_-eq. emissions were from enteric fermentation, manure and soil (directly through denitrification and indirectly through leaching and volatilization) and from feed production and use *i.e.*, for pasture, grass silage and bought-in grain. The methods for calculating GHG emissions are given in the Appendix. 

### 2.5. Data Analysis

The first 10 years of each simulation were discarded to allow the nutrient dynamics within the model to stabilize, leaving a data set from 1971 to 2000 for analysis. Predictions of productivity and emission intensity of sites were obtained with climate, soil type and pasture species as an input to the model. The productivity of each grazing system was assessed by predicting the annual carrying capacity of the pasture system (cow + calf/ha), average annual intake of pasture (t DM ha^−1^) and the average calf live weight at weaning (kg ha^−1^). The productivity of the system was compared to the CO_2_-eq. emissions per ha and per kg calf live weight at weaning. The CO_2_-eq. emissions per kg calf live weight were classified by emissions source: enteric CH_4_, manure and soil GHG, and GHG from feed production (for pasture, grass silage and bought-in grain). 

## 3. Results

### 3.1. Productivity

The average long-term carrying capacity of the pasture at Albany, Dookie, Vasey and Wagga Wagga was predicted to be cows per hectare of 0.6, 0.8, 0.9 and 0.8, respectively. Based on the long-term carrying capacity estimated by the model, simulations were set up to assess the variability in production and GHG emissions of beef cow-calf grazing systems. At each site studied, the predicted peak of average pasture growth during the years 1971 to 2000 was in the spring (September to November) when cows were with their calves (Figure 1). 

The peak of average spring pasture growth was notably greater at Albany with the kikuyu pasture than at other sites studied. Albany also has a temperate climate and is of higher annual rainfall (Table 1). All the grazing systems simulated were predicted to maintain the cow’s daily live weight above 450 kg and ME requirement between 81 to 85 MJ/d (Table 2). 

**Table 2:** Mean (s.d) annual predicted cow live weight and their offspring, total metabolizable energy (ME) requirement, the quality of the diet consumed (ME content and digestibility) and surplus dry matter (DM) cut for beef cow-calf ^1^ grazing systems at Albany, Dookie, Vasey and Wagga Wagga between the years 1971 to 2000 on pasture or a pasture and supplementary feed ^2^ diet.

Site	Units	Pasture	Pasture + supplementary feed
*Albany *	*Kikuyu/subclover pasture and stocking rate 0.6 cows/ha*		
Cow live weight	kg/hd/d	474 (11)	474 (12)
Calf live weight at weaning	kg/hd	96.2 (7.2)	100 (0.2)
ME required	MJ/hd/d	85.3 (1.1)	85.9 (0.5)
Pasture intake	t DM/ha	1.9 (0.2)	1.9 (0.1)
Supplement intake	t DM/ha	0	0.04 (0.05)
ME content	MJ/kg DM	9.6 (0.1)	9.7 (0.1)
DM digestibility	%	60.2 (0.8)	60.3 (0.8)
Pasture cut	t DM/ha	0	1.5 (0.7)
*Dookie*	*Phalaris* */subclover/annual ryegrass pasture and stocking rate 0.8 cows/ha*		
Cow live weight	kg/hd/d	455 (3)	457 (3)
Calf live weight at weaning	kg/hd	62.5 (18)	100 (0.2)
ME required	MJ/hd/d	81.3 (2.1)	86.5 (0.2)
Pasture intake	t DM/ha	0.8 (0.5)	1.0 (0.4)
Supplement intake	t DM/ha	0	0.5 (0.2)
ME content	MJ/kg DM	9.8 (0.3)	10.2 (0.1)
DM digestibility	%	60.9 (1.7)	63.6 (0.6)
Pasture cut	t DM/ha	0	0.3 (0.7)
*Vasey*	*Phalaris* */subclover pasture and stocking rate 0.9 cows/ha*		
Cow live weight	kg/hd/d	456 (1)	457 (1)
Calf live weight at weaning	kg/hd	73.4 (10)	100 (0.2)
ME required	MJ/hd/d	82.5 (1.3)	86.3 (0.1)
Pasture intake	t DM/ha	1.2 (0.3)	1.5 (0.3)
Supplement intake	t DM/ha	0	0.5 (0.1)
ME content	MJ/kg DM	9.9 (0.2)	10.2 (0.1)
DM digestibility	%	61.9 (1.0)	63.5 (0.5)
Pasture cut	t DM/ha	0	0.6 (0.6)
*Wagga* * Wagga*	*Phalaris* */subclover/annual ryegrass pasture and stocking rate 0.8 cows/ha*		
Cow live weight	kg/hd/d	456 (2)	457 (3)
Calf live weight at weaning	kg/hd	74.9 (16)	100 (0.2)
ME required	MJ/hd/d	83.1 (1.8)	86.5 (0.2)
Pasture intake	t DM/ha	0.9 (0.4)	1.1 (0.4)
Supplement intake	t DM/ha	0	0.3 (0.2)
ME content	MJ/kg DM	10.0 (0.3)	10.2 (0.1)
DM digestibility	%	62.4 (1.6)	64.9 (0.7)
Pasture cut	t DM/ha	0	0.6 (0.8)

^1^ A cow over a full year plus its calf from birth until weaning at 120 days of age. ^2^ Grass silage and/or grain.

On average, the total feed intake for a cow + calf per year ranged from 0.8 to 1.9 t DM/ha on pasture and 1.4 to 2.0 t DM/ha on a pasture and supplementary feed diet (Table 2). The use of supplementary feed meant that the target live weight of a calf at weaning of 100 kg was achieved at all sites. However, with no supplementary feed given, the average live weight of a calf ranged from 63 kg at the lower rainfall site of Dookie compared to 96 kg at Albany, which has a higher rainfall and kikuyu pasture.

Table 2 shows that the average annual DM digestibility and ME content of the diet at Albany (which averaged 60% and 9.7 MJ/kg DM respectively across diets) was poorer than at other sites (ranging from 61 to 65% and 9.8 to 10.2 MJ/kg DM, respectively). Surplus pasture was cut to help maintain the availability of new and digestible plant material. 

### 3.2. Greenhouse Gas Emissions and Productivity

Enteric CH_4_ was the main source of emissions from all grazing systems studied (Table 3). The proportion of GEI lost as enteric CH_4_ was predicted to be lower for the kikuyu pasture systems, averaging 7.4% compared to 7.8% for animals on the phalaris based pasture at the other sites. There were small differences between sites and pasture systems in their average CH_4_ produced per kg of digestible dry matter (DDM), which was lowest at Albany (averaging 40.6 g/kg DDM) and slightly higher at the lower rainfall sites of Dookie and Wagga Wagga (averaging 41.7 g/kg DDM). At each site, the main source of N_2_O emissions per hectare was by denitrification, followed by volatilization and then leaching. The emissions of N_2_O by denitrification varied considerably at the lower rainfall sites of Dookie and Wagga Wagga compared to the higher rainfall sites of Albany and Vasey. 

**Table 3:** Annual mean (s.d) carbon dioxide (CO_2_-eq.) equivalent emissions per hectare from enteric methane (CH_4_; expressed as percentage of gross energy intake (GEI) and per digestible dry matter (DDM)), manure CH_4_, nitrous oxide (N_2_O) emissions by leaching, volatilization and denitrification and kilograms of nitrogen fixed by legumes predicted for beef cow-calf ^1^ grazing system at Albany, Dookie, Vasey and Wagga Wagga between the years 1971 to 2000 on pasture or a pasture and supplementary feed ^2^ diet.

Site	Units	Pasture	Pasture + supplementary feed
*Albany*	*Kikuyu/subclover pasture and Stocking rate 0.6 cows/ha*		
Enteric CH_4_	kg CO_2_-eq./ha	926 (68)	944 (51)
Enteric CH_4_	% GEI	7.4 (0.1)	7.4 (0.1)
Enteric CH_4_	g CH_4_/kg DDM	40.6 (0.3)	40.6 (0.3)
Manure CH_4_	kg CO_2_-eq./ha	29.7 (2.7)	30.2 (2.2)
Leaching	kg CO_2_-eq./ha	5.3 (3.1)	5.2 (2.9)
Volatilisation	kg CO_2_-eq./ha	12.6 (2.2)	12.8 (2.5)
Denitrification	kg CO_2_-eq./ha	99.3 (54.7)	100 (56.3)
Legume fixation	kg N/ha	58.4 (18.6)	64.7 (17.2)
Total CO_2_-eq.	t/ha	1.1 (0.1)	1.1 (0.1)
*Dookie*	*Phalaris* */subclover/annual ryegrass pasture and Stocking rate 0.8 cows/ha*		
Enteric CH_4_	kg CO_2_-eq./ha	427 (260)	825 (137)
Enteric CH_4_	% GEI	7.8 (0.1)	7.8 (0.1)
Enteric CH_4_	g CH_4_/kg DDM	42.4 (1.0)	41.2 (0.6)
Manure CH_4_	kg CO_2_-eq./ha	12.6 (7.4)	22.9 (4.3)
Leaching	kg CO_2_-eq./ha	0.1 (0.3)	0.2 (0.3)
Volatilisation	kg CO_2_-eq./ha	5.3 (3.9)	7.9 (2.7)
Denitrification	kg CO_2_-eq./ha	93.9 (110.9)	100 (112)
Legume fixation	kg N/ha	16.4 (6.0)	16.9 (6.5)
Total CO_2_-eq.	t/ha	0.6 (0.4)	0.9 (0.2)
*Vasey*	*Phalaris* */subclover pasture and Stocking rate 0.9 cows/ha*		
Enteric CH_4_	kg CO_2_-eq./ha	645 (148)	1055 (98)
Enteric CH_4_	% GEI	7.8 (0.1)	7.8 (0.1)
Enteric CH_4_	g CH_4_/kg DDM	41.9 (0.5)	40.8 (0.3)
Manure CH_4_	kg CO_2_-eq./ha	18.7 (4.2)	29.7 (3.2)
Leaching	kg CO_2_-eq./ha	1.6 (1.8)	1.8 (2.0)
Volatilisation	kg CO_2_-eq./ha	8.8 (2.8)	12.1 (3.3)
Denitrification	kg CO_2_-eq./ha	42.6 (23.0)	47.0 (23.9)
Legume fixation	kg N/ha	26.2 (7.8)	29.8 (7.2)
Total CO_2_-eq.	t/ha	0.7 (0.2)	1.1 (0.1)
*Wagga* * Wagga*	*Phalaris* */subclover/annual ryegrass pasture and Stocking rate 0.8 cows/ha*		
Enteric CH_4_	kg CO_2_-eq./ha	501 (228)	807 (104)
Enteric CH_4_	% GEI	7.9 (0.1)	7.9 (0.1)
Enteric CH_4_	g CH_4_/kg DDM	42.0 (0.9)	41.2 (0.6)
Manure CH_4_	kg CO_2_-eq./ha	14.1 (6.2)	22.1 (3.2)
Leaching	kg CO_2_-eq./ha	0.001 (0.003)	0.002 (0.004)
Volatilisation	kg CO_2_-eq./ha	6.8 (3.6)	9.2 (2.9)
Denitrification	kg CO_2_-eq./ha	124.3 (97.1)	136 (106)
Legume fixation	kg N/ha	18.0 (6.0)	19.3 (6.6)
Total CO_2_-eq.	t/ha	0.7 (0.3)	1.0 (0.2)

^1^ A cow over a full year plus its calf from birth until weaning at 120 days of age. ^2^ Grass silage and/or grain.

The average annual CO_2_-eq. emissions/ha were 1.1 ± 0.1 t CO_2_-eq. at Albany, 0.6 ± 0.4 and 0.9 ± 0.2 t CO_2_-eq. at Dookie, 0.7 ± 0.2 and 1.1 ± 0.1 t CO_2_-eq. at Vasey, and 0.7 ± 0.3 and 1.0 ± 0.2 t CO_2_-eq. at Wagga Wagga (Table 3) on pasture or pasture with supplementary feed, respectively. Notably, the CO_2_-eq. emissions/ha increased with supplementary feed being given at Dookie, Vasey and Wagga Wagga as the average live weight of a calf at weaning increased (Table 2).

**Figure 2:**
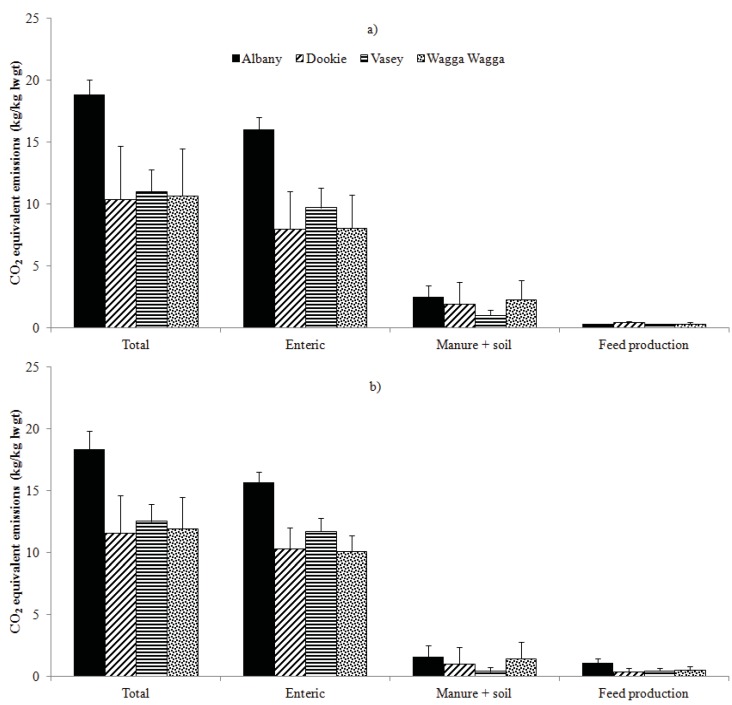
Predicted average annual carbon dioxide (kg CO_2_-eq.) equivalent emissions per kg calf live weight at weaning (kg lwgt) from all sources (total), enteric fermentation, manure and soil, feed production (pasture, forage and bought-in grain) at Albany, Dookie, Vasey and Wagga Wagga for the years 1971 to 2000 for cows fed a) pasture or b) pasture and supplementary feed. Vertical bars indicate standard deviation for 30-year model runs.

Enteric CH_4_ contributed between 0.75 to 0.93, manure and soil between 0.03 to 0.21, and feed production from 0.02 to 0.06 of total CO_2_-eq. emissions per kg calf live weight at weaning across pasture systems (Figure 2). The low stocking rate of cows at Albany compared to their amount of feed consumed contributed to its higher annual enteric CH_4_ emissions per kg calf live weight and CO_2_-eq. emissions per kg calf live weight at weaning. Annual CO_2_-eq. emissions per kg calf live weight at weaning averaged 18.9 ± 1.1 and 18.4 ± 1.5 kg CO_2_-eq. kg^−1^ at Albany, 10.4 ± 4.3 and 11.6 ± 3.0 kg CO_2_-eq. kg^−1^ at Dookie, 11.0 ± 1.8 and 12.5 ± 1.4 kg CO_2_-eq. kg^−1^ at Vasey and 10.7 ± 3.8 and 11.9 ± 2.5 kg CO_2_-eq. kg^−1^ at Wagga Wagga on pasture or pasture with supplementary feed, respectively. 

**Figure 3:**
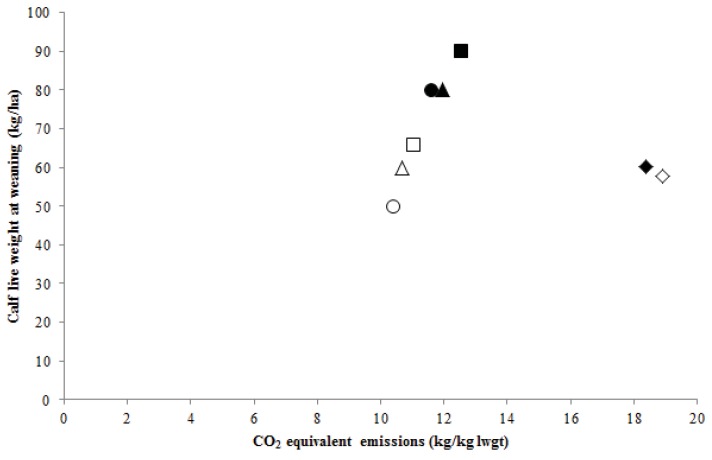
Predicted average annual carbon dioxide (kg CO_2_-eq.) equivalent emissions per kg calf live weight at weaning (kg lwgt) and turnoff of calf live weight at weaning (kg) per hectare at Albany (diamond), Dookie (circle), Vasey (square) and Wagga Wagga (triangle) for the years 1971 to 2000 for cows on pasture (white symbols) or pasture and supplementary feed (black symbols).

As was seen for CO_2_-eq. emissions/ha, the CO_2_-eq. emissions per kg calf live weight increased (Figure 2 and Figure 3) with supplementary feed being given at Dookie, Vasey and Wagga Wagga as the average live weight of a calf at weaning increased (Table 2) and turnoff of calf live weight at each site increased resulting in more enteric CH_4_ emissions per kg calf live weight. Figure 3 shows the CO_2_-eq. emissions per kg calf live weight at Albany showed a slight decline with an increase in productivity with supplementary feed being fed. The use of supplementary feed increased the turnoff of calf live weight at weaning across sites. Due to its higher capacity to carry stock, the phalaris/subclover grazing system at Vasey had the highest predicted total calf live weight at weaning of 66 and 90 kg/ha compared to 60 and 80 kg/ha at Wagga Wagga, 50 and 80 kg/ha at Dookie and 58 and 60 kg/ha at Albany for the pasture or pasture and supplementary feed system, respectively. 

## 4. Discussion

### 4.1. Productivity

Traditionally beef cow-calf systems in southern Australia use minimal inputs and rely on mixed pastures as a low-cost source of energy and nutrients, rather than bought-in feeds. The long-term carrying capacities predicted in this study for Albany, Dookie, Vasey and Wagga Wagga of 0.6, 0.8, 0.9 and 0.8 cows per hectare are comparable to those reported for the Albany site [22] of 0.6 cows per hectare and average values reported [12] for phalaris/subclover pastures at the sites of Vasey and Maindample (in Victoria) of 0.8 to 0.9 cows per hectare. This provided some confidence that the energy requirements of the grazing animal were being appropriately defined in the model.

Of the sites studied, the most productive grazing system was a phalaris/subclover pasture at Vasey. Vasey was predicted to carry a stocking rate of 0.9 cows/ha and produce an average of 66 kg/ha on pasture and 90 kg/ha of calf live weight at weaning on pasture and supplementary feed, compared to 50 to 80 kg/ha across the other sites. The lowest stocking rate modelled was at Albany with 0.6 cows/ha for the kikuyu pasture system. A kikuyu pasture has been found to be productive if the pasture base has a high proportion of legume such as subclover [22]. The kikuyu/subclover pasture was able to fix on average 62 kg N ha^−1^ yr^−1^, which was more than at other sites (ranging from 17 to 28 kg N ha^−1^ yr^−1^ on average). In the study by [22], 40% of the farm was planted with a kikuyu/subclover pasture and the remainder to annual species, which seems appropriate management to utilize the high pasture production of the C4 pasture during the spring and early summer months (September to December in Figure 1) by adjusting livestock numbers to prevent a build-up of less digestible pasture—as occurred in this study with high amounts of pasture cut compared to the phalaris pastures modeled (Table 2). The benefit of kikuyu is that during the warmer months, its deep and adventitious root structure makes it efficient at extracting soil nutrients and water [27,28] and to be productive on deep and freely draining coastal soils such as at Albany [22,29]. Therefore this pasture system can be a useful source of green feed in this location but requires appropriate management to be utilized effectively. A C4 pasture species like kikuyu is better suited to warmer sub-tropical latitudes [30], but may become a more dominant pasture species in the future with warming [10].

### 4.2. Greenhouse Gas Emissions and Productivity

Management, feeding and genetics can all potentially play an important role in reducing system enteric CH_4_ emissions, particularly per unit product [31,32,33]. For all systems modeled, enteric CH_4_ was predicted to be the main source of emissions (0.75 to 0.93 of total system emissions). The annual emissions produced by a cow and its calf in this study (below 75 kg/year at all sites; Table 3) were comparable to published measurements for grazing systems [33]. Given concerns about the quality of C4 dominant pastures compared to those with C3 grasses (such as phalaris) and its impact on enteric CH_4_ emissions [5], this study suggests that a kikuyu pasture system may not necessarily produce more enteric CH_4_ as a percentage of GEI or per kg DDM on average over a year than the C3 pastures studied (7.4% and 40.6 g/kg DDM compared to 7.8% and 41.7 g/kg DDM on average, respectively). The lower enteric CH_4_ lost as a percentage of GEI from a kikuyu grazing system, compared to the C3 systems, could be attributed to the higher level of feed intake per animal. On average the ME content and DM digestibility of the kikuyu pasture system (9.7 MJ/kg DM and 60% respectively) was poorer than the phalaris diets (averaging 9.8 to 10.2 MJ/kg DM and 61 to 65%, respectively). The losses of enteric CH_4_ in this study from a kikuyu pasture were higher than values found by [34] studying grazing dairy cows, who reported measured values for losses of enteric CH_4_ of 7.1% of GEI and 33.8 g/kg DDM. Given the variability in pasture quality, the authors [34] found these values were not that different from a summer perennial ryegrass/white clover pasture and losses of enteric CH_4_ appeared to reduce from the kikuyu pasture when N fertilizer was applied, which improved its DM digestibility. While the model used in this study by [35] to predict enteric methane losses is the accepted method in Australian GHG Inventory, there are concerns that it may over-predict losses from Australian forages [36]. This does not affect comparisons between sites but more the relative contribution of enteric methane to total systems emissions. 

Climate, soil type and management of the pasture species influence the availability of young and digestible plant material, which in turn strongly affects the productivity of the system [5,6]. A study on sheep grazing systems [37] found that management structures and genetic improvements that give the highest sustainable economic yield can also give the lowest enteric CH_4_ emissions intensity per unit product. The average enteric CH_4_ emissions per unit product from the kikuyu pasture system were notably high at 16.1 kg CO_2_-eq. emissions per kg from cows on solely pasture, but declined to 15.7 kg CO_2_-eq. emissions per kg with the addition of supplementary feed, due to the relative level of feed intake to productivity of calf live weight/ha at weaning. At the other sites studied, the average enteric CH_4_ emissions per unit product increased from cows fed pasture and supplement compared to solely grazing pasture, due to an increase in feed intake relative to productivity. In this study and similar to [37], the timing of calving was chosen to coincide with the onset of winter pasture growth, so that pasture supply met the increasing demand from the cow and its calf as well as being of high digestibility and optimizing pasture use (their suggested economic optimum time for parturition). The management of livestock systems can vary considerably, but as found in other studies [37], the efficient use of herbage consumed whilst maintaining optimum animal numbers is important for minimizing grazing system emissions. In this study the grazing system was modeled to provide a digestible pasture by grazing four paddocks and cutting any surplus pasture. Rather than this imposed management on the grazing system, the sites with a more poorly drained chromosol soil type (Dookie and Wagga Wagga) and lower rainfall may benefit from a more flexible grazing management approach e.g., an adjustment in stocking rate. These sites may rely more on supplementary feed to maintain their production of weaned calf or wean calves at a younger age; likely a function of their more variable annual supply of pasture compared to the higher rainfall sites modeled. Developments of the SGS model are required to allow more flexible management options to be assessed. Another limitation of the SGS model is its lack of flexibility in terms of adjusting the number of mature animals with offspring based on the availability of pasture and the sensitivity of the animal to its environment (this is captured more in the growth of the pasture plant). Overall, the use of supplementary feed would contribute a small proportion to total system CO_2_-eq. emissions (0.02 to 0.06). 

The emissions from the grazing system studied could be higher if additional resources were to be sourced from outside the farm unit. As this study suggests and other studies have shown [38,39], pasture-based livestock systems have the potential to be productive and minimize their GHG emissions intensity per unit product and per hectare by being efficient users of natural resources rather than bought-in farm inputs if possible. In comparison to other beef cow production systems which show a wide range of emission intensity, the annual emission intensity per unit product in this study ranged from 10.4 to 18.9 and 11.6 to 18.4 kg CO_2_-eq. emissions kg^−1^ of calf live weight at weaning for pasture or pasture and supplementary feed diets compared to 15.8 to 48.1 kg CO_2_-eq. emissions kg^−1^ of live weight at slaughter for a range of systems in the study of [40] (assuming a dressing percentage of 0.53). In the study by [40] comparing the emission intensity per unit product of beef production, the lowest value reported was for an African pastoral system, whereas the higher intensities were from systems that required higher resource inputs. The feeding of supplement in this study to overcome the constraint of pasture supply did not reduce overall GHG emissions per hectare or emissions per unit product. When pasture supply was adequate and supplement was fed, as simulated in the kikuyu grazing system, a reduction in emissions per unit product was observed with a slight improvement in feed quality and increase in production of calf live weight per hectare at weaning. In beef systems, where enteric CH_4_ is the predominant source of emissions and feed intake is not constrained, improving the feed quality has been found to reduce emissions per unit product by promoting more rapid growth and reducing the number of days to reach a target live weight [41]. This study suggests that there is regional variation in emission intensity per unit product partly caused by differences in soil emissions, feed quality and utilization, which may not necessarily be represented in other studies that do not use location specific inputs to a process-based model. In the present study, the GHG emissions from replacement animals were not included, which can be a significant source of emissions. Another study [42] on beef cow and calf production systems in the southern Australian region included the emissions associated with replacements for a self-replacing breeding system, which as discussed by the authors, inflated the predicted emissions intensity per kg calf live weight (averaging 45.6 kg CO_2_-eq. emissions kg^−1^ of live weight at slaughter) compared to those reported by [42]. 

At the lower rainfall sites of Dookie and Wagga Wagga, variability in manure and soil emissions per ha and per unit product were largely due to N_2_O emissions from denitrification in soil and meant that manure and soil emissions constituted a greater proportion of system emissions on average. The model parameters for simulating nitrogen losses and estimating N_2_O emissions have been compared to measured emissions in a previous study by [43] and were found to provide realistic estimates of N_2_O loses and behavior. The capacity for denitrification within soil is increased with conditions that increase the availability of carbon, temperatures that increase microbial activity and saturation of the WFPS to create anaerobic conditions [44]. Even though more freely draining soils at Albany and Vasey were a greater source of N_2_O leaching losses than at the other sites studied, which was expected [8], losses of N_2_O from leaching and N_2_O losses from N volatilization were less of a source of N_2_O emissions on average compared to denitrification in soil. The differences in N_2_O emissions between sites emphasizes the need for a dynamic modeling approach to accounting for N_2_O emissions from grazing systems in different regions, as opposed to static Tier 1 and 2 emission factors, and would provide a better reflection of these systems in national inventory estimates.

The study used a process-based model to predict both enteric methane and nitrous oxide emissions. As emissions of these gases can be highly variable and are inherently difficult to both measure and model, there are inherent uncertainties in both the model predictions and the measured data sources. Therefore, while the model predictions presented in this study are within plausible boundaries, the emphasis of this study is more on the relative difference between systems modeled at different sites, rather than the absolute values predicted. Process-based models are well suited to predict these relative differences and allow exploration of system behavior.

## 5. Conclusions

In conclusion, while it may not be economically viable, the model simulations of low input grazing systems indicate that the feeding of supplementary feed in the form of grass silage or grain should improve the productivity, but increase CO_2_-eq. emissions per ha. Enteric CH_4_ was the main source of CO_2_-eq. emissions per ha and per unit product. On average, a reduction in enteric CH_4_ emissions per unit product due to the relative level of feed intake to productivity of calf live weight/ha at weaning *i.e.*, a dilution of resource input per unit product, may reduce emission intensity as seen in a supplemented subtropical kikuyu pasture system in this study. By understanding the effect that climatic, edaphic characteristics associated with a particular location can have on GHG emissions, in combination with management decisions, producers could manage their grazing system to maintain low emission intensity per unit product by efficient use of pasture to optimize its carrying capacity.

## Appendix

Emissions of CH_4_ included both enteric fermentation and emissions from manure. Daily enteric fermentation emissions were estimated by the equation developed by Equation (1) [35]:

CH_4_ (MJ d^−1^) = [1.3 + 0.112 × D + FL × (2.37 – 0.05 × D)/100] × GEI
(1)

where D is the digestibility of gross energy (GE) consumed, FL is the feed metabolisable energy (ME) intake relative to that required for maintenance and GEI is the gross energy intake of the animal. The equation of [35] was developed using CH_4_ measurements from sheep and cattle. Equation (1) is currently the accepted method of predicting enteric methane production from cattle in the Australian GHG Inventory [3]. This range for feed intake was greater than the range of possible intakes in this study (from 0 to 16.9 kg). Additionally, the SGS Model predicts DM intake, its digestibility and ME content, which are used to derive the variables in the equation of [35]. The predicted daily DM digestibility of feed consumed was adjusted using the equation of [45] to calculate the digestibility of GE. The proportion of digestible (*δ_dm_*) plant material was calculated by Equation (2) [13]:

*δ_dm_* = *δ_p_f_p_* + *δ_s_f_s_* + *δ_w_f_w_* (2)

where *f* is the content (kg/kg DM) and *δ* is the digestible proportion of plant protein (*p*), sugars (*s*) and cell wall material (*w*), respectively. The model assumed that the contents of plant protein and sugars were completely digestible (*i.e.*, *δ_p_* and *δ_s_* were equal to 1). It was assumed that the cell wall of live plant material had a *δ_w_* of 0.50. All dead plant material had a δ_w_ of 0.2. The ME content (MJ/kg DM) of the pasture was estimated by [13] as *δ_dm_* × 16. The GE content of pasture was assumed to be 18.4 MJ/kg DM [45] and grass silage (based on the findings of [46,47] a 3% higher GE content compared to pasture was assumed) and grain [48] were both 19 MJ/kg DM.

The following assumptions were fixed in the prediction of manure CH_4_ emissions from dung and urine deposited on the pasture based on Australian GHG Inventory values which are: ash content of manure of 0.08 of DM; urinary energy content of manure of 0.04 of GE intake; CH_4_ producing capacity of manure of 0.19 m^3^ kg^−1^ volatile solids; CH_4_ conversion factor of 0.015 [3,49].

Besides CH_4_, the SGS Model apportions predicted N losses (kg N/ha) from the system by volatilisation, denitrification and leaching in this order. This study allocated N from excreta uniformly to the grazing, as [50] showed that it is adequate to assume this type of distribution for stocking rates of less than about 1.5 cows per hectare. It was assumed that 0.01 of the N leached and 0.0125 of the N volatilised was lost indirectly from the system as N_2_O, as used in the Australian GHG Inventory [3]. It is assumed in the SGS Model that ammonia volatilisation from urine was influenced by temperature and rainfall. Temperature had a linear effect on volatilisation (0.20 × kg urine N × temperature °C/20) and rainfall greater than 5 mm stopped volatilisation. Denitrification N_2_O losses were modelled as a function of available soil NO_3_^−^, with the onset of N_2_O losses at 0.6 of the water filled pore space (WFPS), saturation at 0.8 and cessation of N_2_O loss at 0.95 saturation of the WFPS [44,51]. Temperature, WFPS and soil carbon status influenced the rate of denitrification, with a maximum rate of denitrification of 0.25 mg N kg^−1^ soil.

Although it is acknowledged that CO_2_ emissions from soils can be significant, particularly from arable soils [52], following [1] the soil carbon fluxes were assumed to be in a steady state with the amount of respired CO_2_ being equal to the net photosynthesis of the consumed plant matter.

The CO_2_ emissions associated with energy used for managing the pasture, the production of grass silage and the need for supplementary grain were included. It was assumed that the energy required to manage the pasture (for the production, supply and use of machinery and added nutrients) produced 17.64 kg CO_2_-eq. emissions per ha and that the production of 1 kg of grain (wheat) produced 0.4 kg CO_2_-eq. emissions and required 0.16 ha of land to produce [38]. The cutting of surplus grass, its removal or production into grass silage was assumed to produce 32.5 kg CO_2_-eq. t DM^−1^ (as estimated for the harvesting of pasture by [53]), and the fuel required to feed grass silage and grain (using 0.8 litres of fuel per t DM^−1^) produced 3.8862 kg CO_2_-eq. L^−1^ of fuel [54].
